# Phosphorus-32 interstitial radiotherapy for recurrent craniopharyngioma: Expressions of vascular endothelial growth factor and its receptor-2 and imaging features of tumors are associated with tumor radiosensitivity

**DOI:** 10.1097/MD.0000000000011136

**Published:** 2018-06-29

**Authors:** Chenhao Hu, Jinhui Chen, Yuhong Meng, Jianning Zhang, Yaming Wang, Rui Liu, Xin Yu

**Affiliations:** a The Third Clinical College, Southern Medical University, Guangzhou; b Department of Neurosurgery, Navy General Hospital, Beijing, China.

**Keywords:** craniopharyngioma, imaging features, Phosphorus-32 colloid interstitial radiotherapy, VEGF/VEGFR-2

## Abstract

To investigate the relationship of the expression of vascular endothelial growth factor (VEGF)/vascular endothelial growth factor receptor-2 (VEGFR-2) and imaging features with the therapeutic efficacy of Phosphorus-32 colloid interstitial radiotherapy in recurrent craniopharyngioma.

Thirty-two patients with recurrent craniopharyngioma underwent phosphorus-32 colloid interstitial radiotherapy. The tumor imaging features were classified into 4 types according to the thickness of the cyst wall and signals of the cyst contents as shown by computed tomography (CT) and magnetic resonance imaging (MRI) images. Protein expressions of VEGF and VEGFR-2 in craniopharyngioma tissues were evaluated with immunohistochemistry before radiotherapy. The tumor radiosensitivity was determined at 12 months after the interstitial radiotherapy.

VEGF mainly expressed in the tumor cytoplasm, and VEGFR-2 expressed either in vascular endothelial cells or in tumor endothelial cells. VEGF/VEGFR-2 expressions varied significantly in cases sensitive or insensitive to the radiotherapy (VEGF: *P* = .028; VEGFR-2: *P* = .017). Tumor imaging features were associated with the therapeutic efficacy of interstitial radiotherapy (*P* = .000). VEGF expression had no association with the imaging features of tumors (*P* = .226), but VEGFR-2 expression was associated with the imaging features of tumors (*P* = .008).

Our results confirmed the association among imaging features, VEGFR-2 expressions, and tumor radiosensitivity in craniopharyngiomas. Imaging features and VEGFR-2 expressions may add useful data to the radiosensitive assessment of craniopharyngiomas.

## 1 Introduction

Craniopharyngioma is a congenital, invasive epithelial tumor arising from the sellar and the suprasellar region which is classified by histology as benign and accounts for 2% to 5% of primary intracranial tumors.^[9,14,15]^ Surgical resection is the main strategy for treating craniopharyngioma, but only 18% to 84% of tumors can be completely resected surgically because of internal factors, such as tumor location, calcification, range, adhesion, and complex anatomical relationships around the tumor. Additionally, severe complications are commonly seen and postoperative death rate is up to 1.7% to 5.4%. It is confirmed radiologically that the 10-year recurrence rate is 0% to 62% for total tumor resection and 25% to 100% for subtotal or partial removal. Moreover, the difficulty to treat recurrent craniopharyngioma is significantly increased, the surgical resection rate is decreased significantly to 0% to 25%, and the perioperative mortality (10.5–24%) and morbidity are increased significantly.^[4,6,13]^

Stereotactic interstitial radiotherapy has been introduced for treating cystic craniopharyngioma over half a century, with positive efficacy and similar 5- and 10-year overall survival rates to surgical treatment. This therapy is characterized by few complications and low mortality rate, but there are distinct individual differences.^[2,4,10,12]^

Currently, it is a challenge for neurosurgeons that we cannot accurately predict the sensitivity of craniopharyngioma to radiation therapy and relevant influential factors, which is not conducive to accurately and efficiently develop individualized treatment programs. Studies have demonstrated that activities of vascular endothelial growth factor (VEGF) and its receptor-2 (VEGFR-2) are increased in a variety of brain tumors, which not only causes tumor angiogenesis, but also stimulates the proliferation of vascular endothelial cells and tumor cells, thereby exerting an important role in tumor growth, metastasis, and recurrence.^[5,7,11,17,18]^ VEGF/VEGFR-2 active expression in tumor cells and stromal vessels is also relevant to tumor recurrence. However, these studies have focused on the relationship between VEGF/VEGFR-2 and recurrence of craniopharyngioma and angiogenesis, and there is no report on the association between VEGF/VEGFR-2 expression in craniopharyngioma and tumor radiosensitivity. Here, the aim of this study was to evaluate the relationship of imaging features of craniopharyngioma and expression of VEGF/VEGFR-2 in tumor cells with tumor radiosensitivity.

## 2 Materials and methods

### 2.1 Clinical data

This study was a retrospective study that enrolled 32 patients with recurrent craniopharyngioma after first resection from January 2006 to December 2014. There patients consisted of 17 men and 15 women, with an average age of 26.1 years (3–70 years), and 11 of 32 cases (38.9%) were <14 years. Of the 32 cases, there were 29 cases of decreased visual acuity, 27 cases of visual field defects, 6 cases of polydipsia and polyuria, 9 cases of developmental retardation, 6 cases of sexual dysfunction, 7 cases of obesity, 5 cases of electrolyte disorders, and 15 cases of intracranial hypertension. These patients were confirmed to be sensitive or not to stereotactic ^32^P-colloid interstitial radiotherapy that had been performed for 12 months. The study was approved by Ethical committee of Navy General Hospital of PLA and all included patients had signed a general informed consent form.

### 2.2 Tumor classification

**Figure 1:**
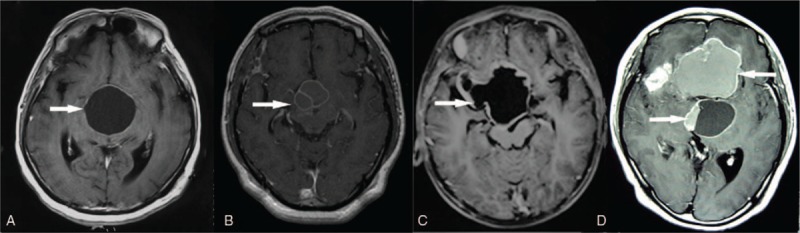
Craniopharyngiomas are classified into 4 types as per pretreatment imaging features. Type I (A); type II (B); type III (C); type IV (D).

According to pretreatment CT and MRI imaging features, the tumors were classified into 4 types. Type I: simple cystic neoplasm with thin cyst wall (CT showed the low-density or equidensity of cyst fluid with no calcification or eggshell calcification, MRI showed homogeneous enhancement or no significant increase in wall thickness [≤1 mm], and the signal intensity of the cyst fluid was long T1 or equal T1 and long T2); type II: the tumors were mainly cystic with solid areas (the solid area accounted for <25% of the entire), single or double cysts were shown with consistent signal components and thin cystic wall (CT showed low-density or equidensity of cyst fluid with eggshell calcification, and significant enhancement of cystic wall was shown on CT and MRI with the wall thickness ≤2 mm, and long T1 and T2 signals); type III: the cyst-based tumor with solid area (the solid area accounted for <25%), single or double cysts were shown with consistent signal components, and thick or irregular wall (CT showed low-density, equidensity or high-density performance of the cyst fluid, and path-like or irregular calcified lesions; the cyst wall thickness was >2 mm or irregular but enhanced significantly on the MRI images, and the cystic fluid showed a variety of T1, T2 signals); type IV: the tumors were partially cystic with solid area, multiple cysts were visible and the cyst fluid showed different signals, or with the cystic liquid–liquid plane (Fig. 1).

### 2.3 ^32^P-colloid interstitial radiotherapy

For radioisotope ^32^P-colloid interstitial radiotherapy, all patients received frame (Leksell-G frame) or frameless (4 marks) stereotactic cyst fluid aspiration (drainage) under local anesthesia, with the exception of patients under 5 years of age who received general anesthesia. Enhanced magnetic resonance imaging (MRI) T1-weighted axial and coronal 2-mm-thick image scans were performed, and the images were transferred to the stereotactic surgery planning system to calculate the tumor volume and determine the target, surgery path, and puncture point. In general, the puncture path should avoid sulci, ventricles, and any nerves and blood vessels. Direct cyst puncture was used to aspirate 1/3 to 1/2 of the cyst fluid, and then the P-32 radioisotope was injected. The colloidal activity of the injected P-32 radioisotope was calculated based on even distribution within the tumor and a 250 Gy prescription dose for the cyst wall. The actual administration activity was determined using nomogram tables as follows: Activity = 0.1365 × (Dose in Gy) × vol (mL)/0.455. These tables are based on a numerical calculation of dose delivered to a surface on the inner wall of a hollow sphere filled with a homogeneous mixture of radiocolloid and water. In all cases, the activity obtained from the nomogram was within 5% of the calculated activity using the previous formula.

### 2.4 Therapeutic efficacy of ^32^P-colloid interstitial radiotherapy and tumor radiosensitivity

At 12 months of interstitial irradiation, enhanced brain MRI was used to calculate tumor volume in comparison with the volume showed on intraoperative MRI. Complete response: the tumor completely disappeared or tumor volume is reduced by >75%; partial response: the tumor volume is reduced by 25% to 75%; stable: the tumor volume is increased or reduced by ≤25%; progress: the tumor volume is increased by >25%. If the tumors completely disappeared or tumor volume is reduced by >50%, the patient can be defined as sensitive to radiation therapy; if the tumor volume is reduced 25% or increased, the patient can be defined as insensitive to radiation therapy.

### 2.5 Immunohistochemical assay

**Figure 2:**
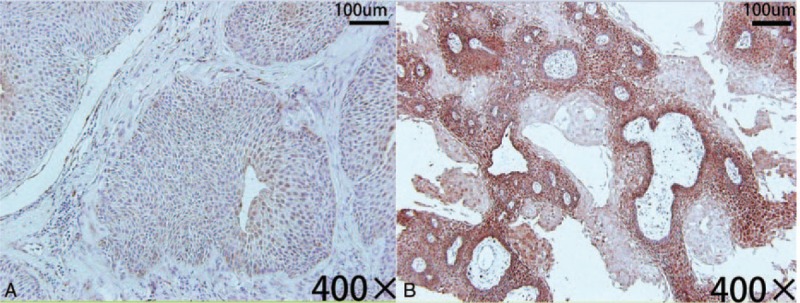
Immunohistochemical staining with anti-VEGFRmAb (original magnification ×400). (A) VEGFR-2 expression was not detected by membranous staining in the lesion. (B) VEGFR-2 expression was obviously detected by membranous staining in the lesion. VEGFR-2 = vascular endothelial growth factor receptor-2.

All tumor specimens obtained from resected tumor tissues were diagnosed as craniopharyngioma by pathologists, and then fixed in 4% neutral formalin, paraffin-embedded, sliced followed by hematoxylin-eosin and immunohistochemical staining. Immunohistochemistry staining was performed using EnVision method, and VEGF/VEGFR-2 anti-mouse monoclonal antibody was purchased from Novocastra (UK). Specimens were developed with DAB, and the operating buffer was TBS. Cells with the presence of brown granules in the cytoplasm or cell membrane were positive for VEGF/VEGFR-2 (Fig. 2). Under 400× magnification, 5 random visual fields per section were selected to calculate the percentage of immunohistochemically positive cells per 200 tumor cells. The coloration of VEGF- and VEGFR-2-positive cells were assessed using semi-quantitative immunohistochemistry method^[16,22]^: negative (–): no positive staining; weakly positive (+): positive cells accounted for <25% or the cells were colored slightly; moderately positive (++): positive cells accounted for 26% to 50%, or were slightly deeply-colored; strong positive (+++): there were >50% positive cells or cells were deeply-colored.

### 2.6 Statistical analysis

Ranked data are expressed as case number and percentage and were analyzed using SPSS19.0 statistical software. Intergroup comparison was tested by a chi-square test, and comparison between multiple groups was tested by a chi-square test followed by the least significant difference test. A *P*-value <.05 was considered statistically significant.

## 3 Results

After 12 months of ^32^P-colloid interstitial radiotherapy, 9 cases were clinically confirmed to be sensitive to the radiotherapy, of which, tumors completely disappeared in 6 cases, and tumor size was reduced >50% in 3 cases; the other 23 cases were defined insensitive to the radiotherapy, of which, the tumor volume was reduced <25% or increased <25% in 9 cases, and increased >25% in 14 cases.

VEGF/VEGFR-2 expression: VEGF mainly expressed in the tumor cytoplasm, and VEGFR-2 expressed either in vascular endothelial cells or in tumor endothelial cells.

Relationship between VEGF/VEGFR-2 expression and pathological types: of the 32 cases, there were 21 cases of adamantinomatouse type (65.6%) and 11 cases of squamous papillary type (34.4%). VEGF/VEGFR-2 expressed in both 2 subtypes, but no significant difference was found (VEGF: *χ*^2^ = 1.542, *P* = .214; VEGFR-2: *χ*^2^ = 3.295, *P* = .348) (Table 1).

**Table 1:**
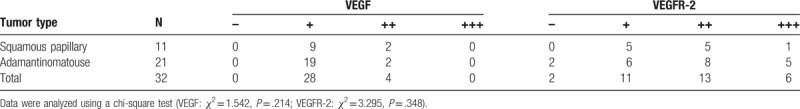
Expression of vascular endothelial growth factor (VEGF) and its receptor (VEGFR-2) in different subtypes of craniopharyngioma.

**Figure 3:**
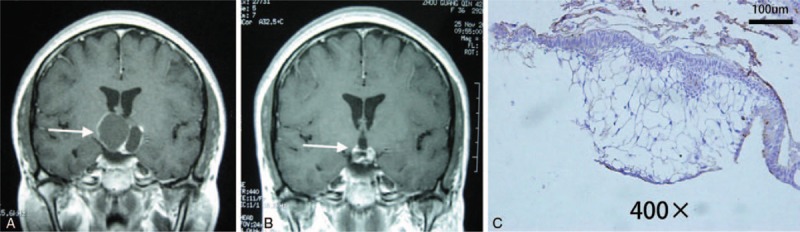
VEGFR-2 expression in craniopharyngioma sensitive to the radiotherapy. (A) Relapse of craniopharyngioma after surgical resection. (B) Tumors basically disappeared at 7 months after ^32^P-colloid interstitial radiotherapy. (C) Tumor specimens were immunohistochemically negative for VEGFR-2 (original magnification ×400). VEGFR-2 = vascular endothelial growth factor receptor-2.

**Figure 4:**
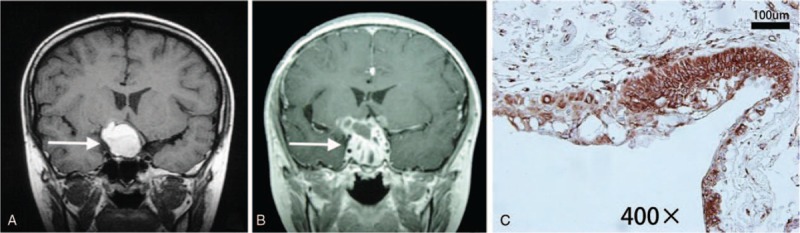
VEGFR-2 expression in craniopharyngioma insensitive to the radiotherapy. (A) Before ^32^P-colloid interstitial radiotherapy. (B) The tumor was enlarged in size and solidified at 12 months after ^32^P-colloid interstitial radiotherapy. (C) VEGFR-2 was extremely expressed in tumor specimens (original magnification ×400). VEGFR-2 = vascular endothelial growth factor receptor-2.

Relationship between VEGF/VEGFR-2 expression and tumor radiosensitivity: VEGF/VEGFR-2 expression differed significantly in cases sensitive or insensitive to the radiotherapy (VEGF: *χ*^2^ = 4.429, *P* = .035; VEGFR-2: *χ*^2^ = 9.558, *P* = .023) (Table 2). Two cases with no VEGF/VEGFR-2 expression were confirmed sensitive to the radiotherapy (Fig. 3), and 6 patients with extremely high VEGFR-2 expression were non-sensitive to the radiotherapy (Fig. 4).

**Table 2:**
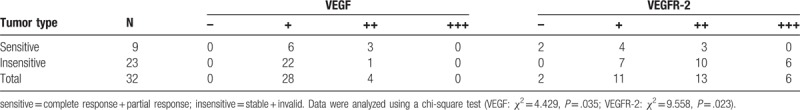
Expression of vascular endothelial growth factor (VEGF) and its receptor (VEGFR-2) in craniopharyngioma sensitive or insensitive to radiotherapy.

Relationship between imaging features of tumors and efficacy of radiotherapy: tumor imaging features were associated with therapeutic efficacy of interstitial radiotherapy (*χ*^2^ = 18.124, *P* = .000). Four imaging features of tumors types were compared in pairs, and the results demonstrated the curative effect of type I and II craniopharyngioma are better than type III and IV craniopharyngioma (Tables 3 and 4).

**Table 3:**
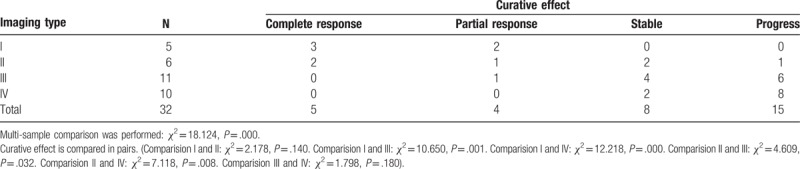
Association between imaging features of craniopharyngioma and curative effect of interstitial radiotherapy.

**Table 4:**
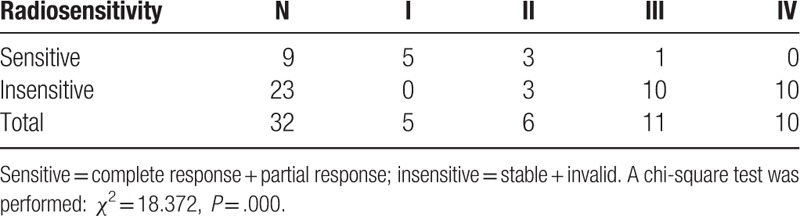
Association between imaging features of craniopharyngioma and tumor radiosensitivity.

Relationship between VEGF/VEGFR-2 expression and imaging features of tumors: the imaging features of tumors were associated with VEGFR-2 (*χ*^2^ = 24.555, *P* = .004) rather than VEGF (*χ*^2^ = 5.194, *P* = .158). Expression of vascular endothelial growth factor (VEGF) and its receptor (VEGFR-2) in craniopharyngioma with different imaging features were also compared in pairs, and the result verified that the type IV craniopharyngioma show the negative rate of VEGFR-2 is lower than others, and the positive rate is higher than others (Table 5).

**Table 5:**
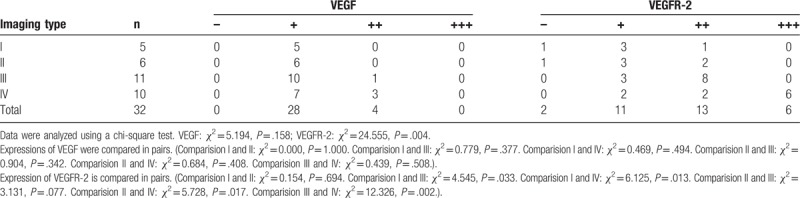
Expression of vascular endothelial growth factor (VEGF) and its receptor (VEGFR-2) in craniopharyngioma with different imaging features.

These findings indicate that patients with type I and II craniopharyngioma who have low or no expression of VEGFR-2 in tumor endothelial cells are sensitive to the ^32^P-colloid interstitial radiotherapy; patients with type III and IV craniopharyngioma show a high expression of VEGFR-2 in tumor endothelial cells with no sensitivity to the ^32^P-colloid interstitial radiotherapy.

## 4 Discussion

Radiotherapy that includes fractionated radiation, interstitial radiation, and gamma knife therapy is the primary therapy for craniopharyngioma, and additionally, it is also an important auxiliary and complementary therapy for surgical treatment.^[1,9,10]^ However, some patients have no satisfied outcomes because of the presence of radiation-resistant tumor cells that leads to uncertain choice of treatment methods and prognostic evaluation.^[23,24]^ The biggest problems in the treatment of craniopharyngioma are shown as follows: to decide which patients will be subjected to aggressive treatment and which patients are suitable for radiotherapy, to determine the optimal therapeutic scheme for a particular patient, and to expect the treatment outcomes. Therefore, it is of great significance to investigate the influential factors of tumor radiosensitivity to determine the choice of individualized treatment and improve the curative efficacy in patients with craniopharyngioma.^[3,14,25,26]^

Craniopharyngioma is classified by histopathology into adamantinomatouse type and squamous papillary type, and it is also classified by morphology into solid, cystic and mixed. Cystic tumors include single and polycystic types.^[9,14]^ In the present study, the tumors were classified into 4 types as per pretreatment imaging features (types I, II, III, IV). This classification focused on the thickness of the cystic wall, components of the cyst fluid (slight or pale yellow, deep yellow, dark yellow, brown or color of soy sauce) and whether the components within the cysts are consistent. Generally, type I is mostly defined as slight or pale yellow, type II as deep yellow, type III as brown, type IV as color of soy sauce and inconsistent color of the cyst fluid (indicating tumor components are more complex).^[4,7,27]^ Our results showed that types I and II were mostly sensitive to the ^32^P-colloid interstitial radiotherapy, and types III and IV were mostly insensitive to the ^32^P-colloid interstitial radiotherapy. This means that: tumors with thin cystic wall and consistent signals of the intracystic components were sensitive to the interstitial radiotherapy, while those with thick cystic wall or uniform signals were insensitive to the interstitial radiotherapy. These findings show that the imaging features of craniopharyngioma are associated with the curative effect of the ^32^P-colloid interstitial radiotherapy, indicating different cyst fluid composition and secreted form of tumor cells can influence the curative effect of interstitial radiotherapy. However, the specific mechanism and the effect on tumor radiosensitivity need to be explored.^[7,26,27]^

As previously reported,^[3,8]^ the radiosensitivity of brain malignant tumors and craniopharyngioma is directly associated with tumor angiogenesis, which is consistent with studies on other in vivo tumors. Tumor angiogenesis is crucial for the occurrence and development of tumors, which is a delicate balance between stimulating and inhibiting the proliferation of endothelial cells.^[3,8,28]^ VEGF secreted by most tumors is the most important inducing factor for tumor angiogenesis, and its receptor (VEGFR-2) is mainly expressed in the vascular endothelial cells to mediate endothelial cell division and to influence vascular permeability.^[16,32]^ VEGF/VEGFR-2 expression is also associated with the development of benign intracranial tumors. For example, VEGF expression is associated with the hemorrhage, cystic degeneration, and malignant invasion of pituitary adenoma.^[7,16,22]^ Additionally, VEGF mainly expresses in the nests of craniopharyngioma cells (cytoplasm and matrix), and shows a higher level in the recurrent craniopharyngioma patients, indicating VEGF plays a vital role in the invasive growth and recurrence of craniopharyngioma.^[19]^ In recent years, VEGFR-2 is found to express not only in vascular endothelial cells, but also in some non-endothelial cells, such as melanocytes, retinal epithelial cells, hematopoietic stem cells, neuronal cells, and some tumor cells (glioblastoma, hemangioblastoma, and meningioma).^[5,11,20,21]^ Vidal et al^[18]^ reported that VEGFR-2 mRNA is expressed not only in interstitial capillaries, but also in the epithelium of craniopharyngioma. These findings indicate an increased possibility that VEGF produces an effect on the epithelial components, and it is also confirmed that the VEGFR-2 acts as the important regulator for the activity of VEGF in endothelial and non-endothelial cells.^[8]^ Findings from the present study show that if the VEGFR-2 has low or no expression in craniopharyngioma, the tumors are mostly defined radiographically as type I and II, and sensitive to the ^32^P-colloid interstitial radiotherapy; if the VEGFR-2 has a high expression in craniopharyngioma, the tumors are mostly defined radiographically as type III and IV, and insensitive to the ^32^P-colloid interstitial radiotherapy.^[4,33,34]^ This is probably because the ^32^P-colloid interstitial radiotherapy not only directly damages the tumor cells, but also activates the VEGF/VEGFR-2 system to promote tumor angiogenesis and to vary the tumor microenvironment, thereby resulting in tumor insensitivity to the radiotherapy. In addition, we found that 2 cases were insensitive to radiotherapy, in which, VEGFR-2 was only expressed in the endothelial cells of craniopharyngioma rather than in the vascular endothelial cells. VEGF/VEGFR-2 may play an important role in the regulation of tumor cell proliferation and insensitivity to radiotherapy by activating mitogen-activated protein kinase signaling pathway and subsequent mitogenic responses.^[5,20]^ Accordingly, we hypothesized that VEGF/VEGFR-2 signals are involved in the regulation of radiotherapy resistance in the treatment of craniopharyngioma. Another possibility is that the activation of VEGF/VEGFR-2 may result in increased permeability of epithelial cells and cyst formation.

Our findings demonstrate that imaging features of craniopharyngioma and VEGF/VEGFR-2 expression in tumor cells are associated with tumor radiosensitivity. If thin cystic wall and consistent intracystic signals (type I and II) are shown on CT and MRI, and VEGFR-2 is expressed lowly or not expressed in tumor endothelial cells, the tumors are mostly sensitive to the ^32^P-colloid interstitial radiotherapy; otherwise, if thick cystic wall and inconsistent intracystic signals are shown on CT and MRI, and VEGFR-2 is highly expressed in tumor endothelial cells, the tumors are mostly insensitive to the ^32^P-colloid interstitial radiotherapy. If this hypothesis is further confirmed, this study will provide theoretical evidence for guiding the selection of individualized treatment for recurrent craniopharyngioma, especially the choice of interstitial radiotherapy or conventional external radiotherapy, as well as prognostic evaluation.

## 5 Conclusion

Imaging features and VEGFR-2 expressions of craniopharyngioma are associated with tumor radiosensitivity. Low VEGFR-2 expression, <2 mm cyst wall and homogeneous low-density in imaging imply higher radiosensitivity for craniopharyngioma. On the contrary, high VEGFR-2 expression, >2 mm cyst wall and uneven density in imaging indicate the craniopharyngioma patients are not sensitivity to the ^32^P-colloid interstitial radiotherapy. Imaging features and VEGFR-2 expressions may add useful data to the radiosensitive assessment of craniopharyngiomas.

## Author contribution

**Conceptualization:** Xin Yu.

**Data curation:** Jianning Zhang, Xin Yu.

**Formal analysis:** Jinhui Chen, Yaming Wang.

**Investigation:** Xin Yu.

**Methodology:** Yuhong Meng, Xin Yu.

**Project administration:** Yaming Wang.

**Resources:** Rui Liu.

**Software:** Rui Liu, Xin Yu.

**Writing – review and editing:** Chenhao Hu.
